# Reference formulas for chest CT-derived lobar volumes in the lung-healthy general population

**DOI:** 10.1007/s00330-024-11123-6

**Published:** 2024-10-16

**Authors:** Jens T. Bakker, Ivan Dudurych, Sharyn A. Roodenburg, Judith M. Vonk, Karin Klooster, Marleen de Bruijne, Maarten van den Berge, Dirk-Jan Slebos, Rozemarijn Vliegenthart

**Affiliations:** 1 Department of Pulmonary Diseases, University of Groningen, University Medical Center Groningen, Groningen, The Netherlands; 2 Groningen Research Institute for Asthma and COPD (GRIAC), University of Groningen, University Medical Center Groningen, Groningen, The Netherlands; 3 Department of Radiology, University of Groningen, University Medical Center Groningen, Groningen, The Netherlands; 4 Department of Epidemiology, University of Groningen, University Medical Center Groningen, Groningen, The Netherlands; 5 Department of Radiology and Nuclear Medicine, Erasmus MC–University Medical Center Rotterdam, Rotterdam, The Netherlands; 6 Department of Computer Science, Copenhagen University, Copenhagen, Denmark

**Keywords:** Lung, Pulmonary disease (chronic obstructive), Tomography (X-ray computed), Lung volume measurements, Population

## Abstract

**Introduction:**

Lung hyperinflation, a key contributor to dyspnea in chronic obstructive pulmonary disease (COPD), can be quantified via chest computed tomography (CT). Establishing reference equations for lobar volumes and total lung volume (TLV) can aid in evaluating lobar hyperinflation, especially for targeted lung volume reduction therapies.

**Methods:**

The Imaging in Lifelines study (ImaLife) comprises 11,729 participants aged 45 and above with analyzed inspiratory low-dose thoracic CT scans. Lung and lobar volumes were measured using an automatic AI-based segmentation algorithm (LungSeg). For the main analysis, participants were excluded if they had self-reported COPD/asthma, lung disease on CT, airflow obstruction on lung function testing, were currently smoking, aged over 80 years, or had height outside the 99% confidence interval. Reference equations for TLV and lobar volumes were determined using linear regression considering age and height, stratified by sex. For the subanalysis, participants who were currently smoking or experiencing airflow obstruction were compared to the group of the main analysis.

**Results:**

The study included 7306 lung-healthy participants, 97.5% Caucasian, 43.6% men, with mean age of 60.3 ± 9.5 years. Lung and lobar volumes generally increased with age and height. Men consistently had higher volumes than women when adjusted for height. *R*^2^ values ranged from 7.8 to 19.9%. In smokers and those with airway obstruction, volumes were larger than in lung-healthy groups, with the largest increases measured in the upper lobes.

**Conclusion:**

The established reference equations for CT-derived TLV and lobar volumes provide a standardized interpretation for individuals aged 45 to 80 of Northern European descent.

**Key Points:**

***Question*** *Lobar lung volumes can be derived from inspiratory CT scans, but healthy-lung reference values are lacking.*

***Findings*** *Lung and lobar volumes generally increased with age and height. Reference equations for lung/lobar volumes were derived from a sizeable lung-healthy population.*

***Clinical relevance*** *This study provides reference equations for inspiratory CT-derived lung and lobar volumes in a lung-healthy population, potentially useful for assessing candidates for lung volume reduction therapies, for lobe removal in lung cancer patients, and in case of restrictive pulmonary diseases.*

## Introduction

Chronic obstructive pulmonary disease (COPD) is a debilitating condition characterized by the inability to effectively exhale. This can result in a state of hyperinflation where more air is retained in the lungs compared to individuals without disease. In clinical practice, body plethysmography is used to quantify the severity of total hyperinflation through the measurement of static lung volumes, especially total lung capacity (TLC) and residual volume (RV). To aid the interpretation of these measurements, the absolute volumes are standardized using reference values that are based on individuals without lung disease and are similar in terms of height, age, and sex, the most recent being established by the Global Lung Initiative (GLI) [1, 2].

Accurate assessment of lung volumes is crucial to select appropriate patients for interventions targeting hyperinflation, like endobronchial valve (EBV) treatment. This treatment aims to reduce lung volume by inducing a lobar atelectasis. Selecting the correct target lobe is essential because an inaccurate selection may exacerbate the patient’s condition rather than provide relief from symptoms. Generally, the lobe of interest is the one most severely affected by emphysematous destruction, which therefore contributes little to gas exchange [3]. Additional considerations include fissure completeness to prevent collateral ventilation and balancing the target lobe volume with the ipsilateral lobar volume(s) [4]. For these reasons, computed tomography (CT) scanning and quantitative CT analysis by lobar segmentation are essential for patient selection for EBV treatment, as no other modality can provide accurate lobar-based information. However, unlike plethysmography-derived volumes, standardized reference values for lobar volumes have not been universally established. Only one study has derived such equations, albeit in a relatively small cohort [5]. This kind of information could be valuable for assessing the feasibility of EBV treatment, determining, for example, whether hyperinflation is confined to a specific lobe.

Several studies have demonstrated that lung volumes can be established by CT, with a strong correlation to their plethysmography counterparts [6–13]. We will refer to the CT-derived equivalent of TLC as total lung volume (TLV). One study highlighted that the repeatability of CT-derived volumes surpasses that of plethysmography-derived volumes [11]. We have recently demonstrated that lung volumes compare well to plethysmography counterparts in COPD patients as well, especially when spirometry-gating is used [14], highlighting the potential utility in EBV treatment assessment. Despite high correlations between both modalities, relying on GLI-predicted reference values for TLC in CT-derived TLV measurements is inadequate as the GLI equations significantly overestimate actual TLV values in a lung-healthy population [15].

Thus, while lung volumes can be accurately measured using CT, proper reference equations for normal values are lacking. We aim to establish reference equations, using age, sex and height in a similar fashion to the GLI model, for CT-derived lobar volumes and TLV in a lung-healthy cohort, representative of the Northern European general population.

## Methods

### Population

The Imaging in Lifelines study (ImaLife) comprises 11,762 participants aged 45 and above with an assessable inspiratory low-dose thoracic CT scan. ImaLife is part of the larger LifeLines cohort study. Lifelines is a multi-disciplinary prospective population-based cohort study examining, in a unique three-generation design, the health and health-related behaviors of 167,729 persons living in the north of the Netherlands, with 97.5% of them having Caucasian origin. It employs a broad range of investigative procedures in assessing the biomedical, socio-demographic, behavioral, physical and psychological factors that contribute to the health and disease of the general population, with a special focus on multi-morbidity and complex genetics [16]. As a part of this, ImaLife is focused on imaging biomarkers that are gathered from low-dose chest CT scans. Lifelines participants aged 45 or older who had lung function test data available for the second visit in the Lifelines study were invited to undergo low-dose inspiratory chest CT scanning. The images were reviewed by trained radiologists(-in-training) or a trained technical medicine doctor under the supervision of radiologists. The study design has been described in a previous publication [17].

The current study consists of two parts: the main analysis and the subanalysis. The focus of the main analysis was on the lung-healthy population. We excluded participants with airflow obstruction as indicated by a Forced Expiratory Volume in 1 s (FEV_1_) to Forced Vital Capacity (FVC) ratio below the lower limit of normal in spirometry, current smoking, self-reported history of obstructive pulmonary diseases (COPD/asthma), signs of lung disease on CT, individuals with a height outside the 99% confidence interval (CI) to address extreme outliers, and finally, participants aged over 80 years due to insufficient sample size. Specifics on exclusion criteria can be found in the Supplement. Furthermore, for the subanalysis, the participants with airflow obstruction and with current smoking were included. The exact methods are described below.

This study has been approved by the medical ethics committee of the University Medical Center Groningen, the Netherlands, and has been registered with the Dutch Central Committee on Research involving Human Subjects (https://www.toetsingonline.nl, Identifier: NL58592.042.16). All participants provided written informed consent.

### Image acquisition, reconstruction and analysis

Each participant underwent a supine, breath-coached to inspiration, low-dose chest CT scan using a third-generation dual-source CT scanner (SOMATOM Force, Siemens Healthineers). The scans were performed between August 2017 and October 2022. The scan was reconstructed using a sharp Qr59 kernel specifically designed for quantitative analysis, and a slice thickness of 1 mm and a slice increment of 0.7 mm. All scans were automatically processed using an automatic AI-based lung and lobe segmentation algorithm called LungSeg (https://github.com/jtabalon/LungQuant) [18]. These segmentations were used to calculate the total inspiratory lung volume (TLV) and lobar volumes. Volume was calculated by multiplying the number of voxels in each segmentation by the volume of an individual voxel. Segmentations with a TLV greater than 8 L or less than 3 L were flagged for visual inspection by a trained medical doctor (I.D.). Abnormal segmentations were subsequently removed from the dataset.

### Statistical analysis

The TLV and lobar volumes were visually assessed for a normal distribution. The reference equations were obtained by linear regression. Analogously to the reference equations established by GLI, this regression was stratified for sex, and the independent variables were age and height [2]. Statistical analyses and visualizations were conducted using R version 4.2.2 (R Core Team). *p*-values below 0.05 were considered significant. The accuracy of the reference equations was evaluated using the residual standard deviation (RSD), which measures the standard deviation of the differences between the predicted and observed values. Additionally, the adjusted regression coefficient squared (*R*^2^) was used to assess the strength of the correlation between the reference equations and the observed values. Expressed as a percentage, *R*^2^ indicates the proportion of variance in the observed data that is explained by the model.

### Subanalysis of subgroups with lung health impairment

To investigate if the volumes in current smokers and people with airway obstruction (FEV_1_/FVC ratio below the lower limit of normal) are indeed larger than in the lung-healthy group, we applied the calculated reference equations to current smokers and people with airway obstruction and compared the percentage predicted values in these groups to the values in the lung-healthy group. The current smokers and individuals with airway obstruction groups were made up of participants who were excluded from the lung-healthy group and were screened for all other exclusion criteria (including current smoking for airway obstruction group, and airway obstruction for current smoking group). We conducted independent two-sided *t*-tests, with Bonferroni correction applied to mitigate the effects of multiple comparisons, to compare the lung and lobar volumes of both these groups against those of the lung-healthy population.

## Results

### Population

**Fig. 1:**
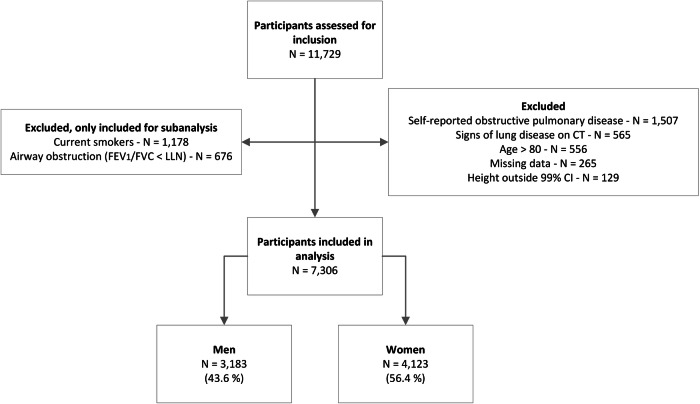
Flowchart of patient selection. CI, confidence interval; LLN, lower limit of normal; *N*, number of individuals

The participant selection process is illustrated in Fig. 1. For all participants (*N* = 11,729), the inspiratory CT scan was analyzed using LungSeg. A total of 1343 (11.5%) patients exhibited a FEV_1_/FVC ratio below the lower limit of normal, 1265 (10.8%) were identified as current smokers, 898 (7.7%) reported a history of pulmonary disease (COPD or asthma), 356 (3.0%) displayed signs of lung disease on CT, 266 (2.3%) participants were over the age of 80, 166 (1.4%) had missing data, and 129 (1.1%) had a height outside the 99% confidence interval. Ultimately, 7306 participants were included and stratified by sex. Among them, 3183 (43.6%) were men and 4123 (56.4%) were women. Men had a mean age of 61.0 ± 9.6 years and a height of 1.82 ± 0.07 m, while women had a mean age of 59.7 ± 9.4 years and a height of 1.68 ± 0.06 m. Men and women demonstrated a significant difference for all considered parameters according to independent two-sided *t*-tests. Further demographic details of the included participants are presented in Table 1.

**Table 1:** Demographic characteristics of the study population

	Men (*n* = 3183)	Women (*n* = 4123)	*p*-value
**Characteristics**			
Age, years	61.0 ± 9.6	59.7 ± 9.4	< 0.001
Height, m	1.82 ± 0.07	1.68 ± 0.06	< 0.001
Weight, kg	86.9 ± 12.4	73.8 ± 12.6	< 0.001
**Spirometry**			
FEV_1_, L	3.92 ± 0.65	2.84 ± 0.47	< 0.001
FEV_1%_-predicted, %	103.6 ± 13.5	105.6 ± 13.7	< 0.001
FVC, L	5.17 ± 0.82	3.71 ± 0.59	< 0.001
FVC %-predicted, %	107.1 ± 14.1	108.2 ± 14.1	< 0.001
FEV_1_/FVC ratio	0.76 ± 0.05	0.77 ± 0.05	< 0.001
**CT-derived volumes**			
TLV (L)	6.12 ± 1.20	4.71 ± 0.79	< 0.001
Volume LUL (L)	1.47 ± 0.28	1.09 ± 0.18	< 0.001
Volume LLL (L)	1.37 ± 0.37	1.08 ± 0.25	< 0.001
Volume RUL (L)	1.19 ± 0.23	0.89 ± 0.16	< 0.001
Volume RML (L)	0.56 ± 0.13	0.40 ± 0.09	< 0.001
Volume RLL (L)	1.51 ± 0.40	1.24 ± 0.27	< 0.001

The demographics are summarized as follows: Data is expressed as the mean ± standard deviation for continuous variables, while participant numbers are presented as counts and percentages relative to the total within each group. *p*-values were determined using an independent two-sided *t*-test

*FEV1* forced expiratory volume in 1 s, *FVC* forced vital capacity, *TLV* total lung volume, *LUL* left upper lobe, *LLL* left lower lobe, *RUL* right upper lobe, *RLL* right lower lobe, *RML* right middle lobe

### Regression analysis in lung-healthy population

**Fig. 2:**
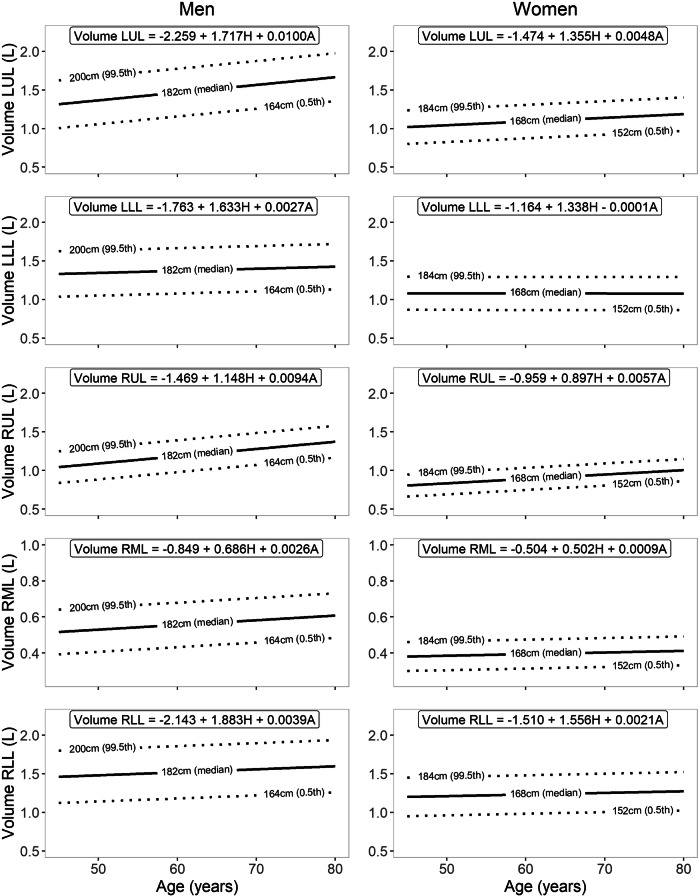
Reference equations for lobar volumes at all included ages for men and women for median, 0.5 percentile and 99.5 percentile heights. LUL, left upper lobe; LLL, left lower lobe; RUL, right upper lobe; RLL, right lower lobe; RML, right middle lobe

**Fig. 3:**
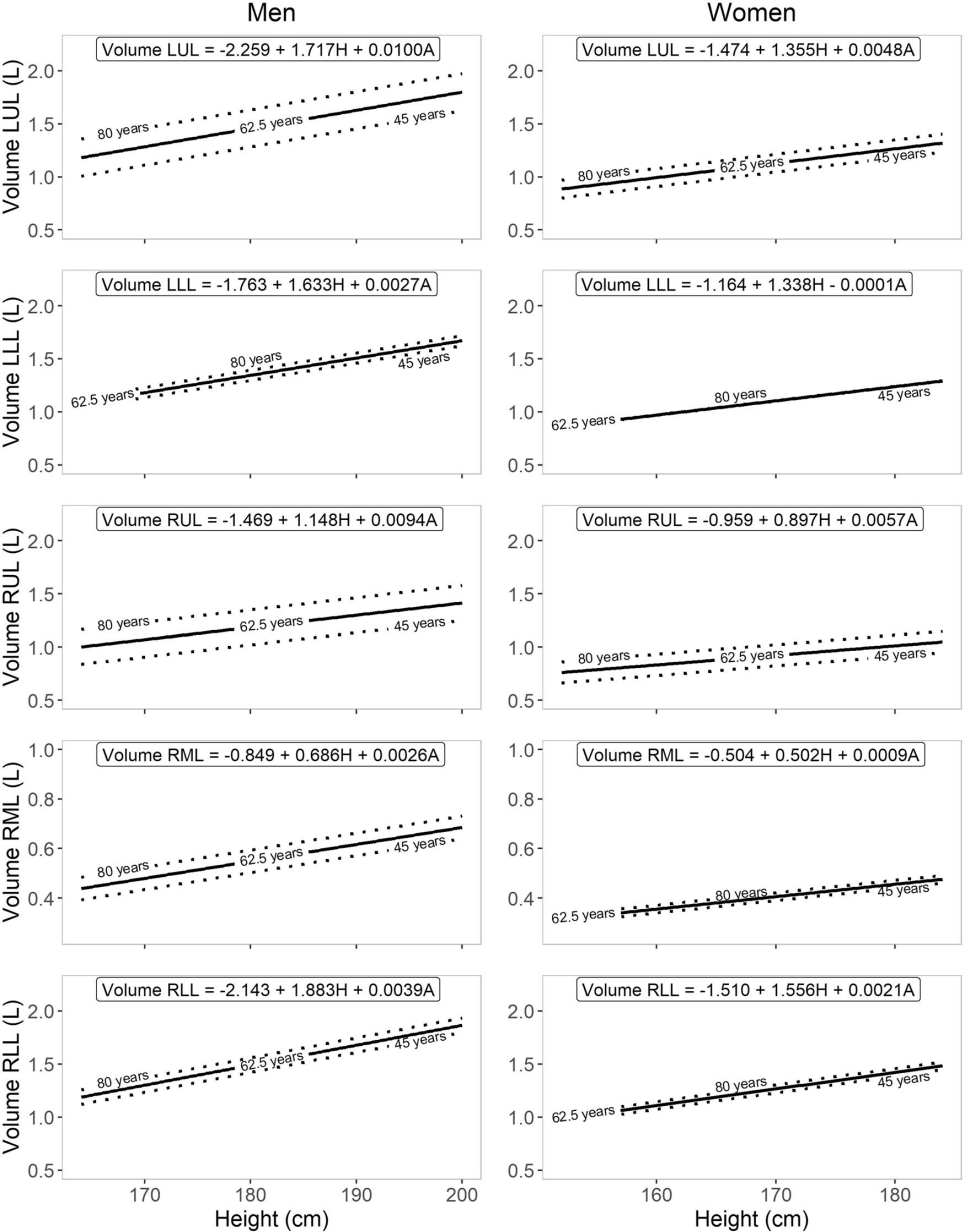
Reference equations for lobar volumes from the minimum included height to the maximum included height for men and women for the minimum age, around the median age and the maximum age. LUL, left upper lobe; LLL, left lower lobe; RUL, right upper lobe; RLL, right lower lobe; RML, right middle lobe

All volumes were normally distributed. Men demonstrated higher lung and lobar volumes at the same height than women. The regression models fitted for the volumes demonstrated consistent patterns, particularly positive associations with both age and height, with the exception of the LLL in women, as this volume did not seem to be associated with age. The explained variance by the regression models ranged from 7.8 to 19.9%. In general, the RLL was the largest lobe, followed by the LUL and the LLL, which were about equal in size. The RUL was smaller, and the RML was, by a large margin, the smallest. The volume of the upper lobes was more related to age than the lower lobes; with increasing age, the upper lobes comprised an ever-larger proportion of the TLV for both men and women. At advanced age, this led to the LUL, on average, becoming larger than the RLL in men. The reference equations can be appreciated in Table 2. Figure 2 demonstrates the volumes for increased age at three different heights. Figure 3 demonstrates the volumes for increasing height at three ages.

**Table 2:** Regression analysis with the resulting reference equations

Parameter	Reference equation	RSD	*R*^2^
**Men**			
TLV (L)	−8.514 + 7.090 × H + 0.0287 × A	1.107	0.151
Volume LUL (L)	−2.259 + 1.717 × H + 0.0100 × A	0.251	0.199
Volume LLL (L)	−1.763 + 1.633 × H + 0.0027 × A	0.354	0.078
Volume RUL (L)	−1.469 + 1.148 × H + 0.0094 × A	0.213	0.179
Volume RML (L)	−0.849 + 0.686 × H + 0.0026 × A	0.123	0.116
Volume RLL (L)	−2.143 + 1.883 × H + 0.0039 × A	0.382	0.087
**Women**			
TLV (L)	−5.626 + 5.664 × H + 0.0133 × A	0.713	0.177
Volume LUL (L)	−1.474 + 1.355 × H + 0.0048 × A	0.164	0.199
Volume LLL (L)	−1.164 + 1.338 × H − 0.0001 × A	0.236	0.107
Volume RUL (L)	−0.959 + 0.897 × H + 0.0057 × A	0.144	0.167
Volume RML (L)	−0.504 + 0.502 × H + 0.0009 × A	0.088	0.098
Volume RLL (L)	−1.510 + 1.556 × H + 0.0021 × A	0.251	0.116

*RSD* residual standard deviation, *R*^*2*^ adjusted regression coefficient squared, *TLV* total lung volume, *LUL* left upper lobe, *LLL* left lower lobe, *RUL* right upper lobe, *RLL* right lower lobe, *RML* right middle lobe, *H* height (m), *A* age (years)

### Populations with lung health impairments

The mean percentage predicted TLV using the reference equations of Table 2 was 103.1 ± 17.5% for the current smoking subgroup, and 110.3 ± 16.9% for the airway obstruction subgroup. Table 3 demonstrates these values in addition to the mean percentage predicted values with standard deviations for all the lobar volumes within current smokers and airway obstruction subgroups, all of which follow normal distributions. The percentage predicted values of the lung and lobar volumes of the airway obstruction subgroup were significantly different from the lung-healthy population (all *p* < 0.001). The male current smokers were significantly different from the lung-healthy population regarding percentage predicted values for TLV (*p* = 0.002), LUL and RUL (both *p* < 0.001), but not for LLL (*p* = 0.835), RML (*p* = 1) and RLL (*p* = 0.410). For female current smokers, all lung and lobar predicted volumes were significantly different from the lung-healthy population (all *p* < 0.001, except LLL: *p* = 0.025), with the exception of the RLL (*p* = 1). The largest differences were observed in the upper lobes.

**Table 3:** Percentage of predicted lung and lobar volumes across current smokers, and airway-obstructed groups

	Current smokers	Current smokers versus lung-healthy Adjusted *p*-value	Airway obstruction	Airway obstructed versus lung-healthy Adjusted *p*-value
**Men**				
Participants, *N*	603		298	
TLV, % predicted	103.1 ± 17.5	**0.002**	110.3 ± 16.9	**< 0.001**
LUL, % predicted	103.4 ± 17.3	**< 0.001**	110.1 ± 17.7	**< 0.001**
LLL, % predicted	102.5 ± 25.9	0.835	110.4 ± 25.5	**< 0.001**
RUL, % predicted	105.2 ± 17.8	**< 0.001**	113.0 ± 21.6	**< 0.001**
RML, % predicted	101.1 ± 21.7	1	107.6 ± 25.8	**< 0.001**
RLL, % predicted	102.6 ± 23.9	0.410	109.2 ± 23.9	**< 0.001**
**Women**				
Participants, *N*	575		398	
TLV, % predicted	103.1 ± 15.3	**< 0.001**	108.0 ± 14.7	**< 0.001**
LUL, % predicted	103.2 ± 15.1	**< 0.001**	108.4 ± 16.4	**< 0.001**
LLL, % predicted	103.2 ± 22.0	**0.025**	108.2 ± 22.0	**< 0.001**
RUL, % predicted	104.2 ± 16.9	**< 0.001**	110.1 ± 18.7	**< 0.001**
RML, % predicted	104.4 ± 22.2	**< 0.001**	104.8 ± 25.9	**< 0.001**
RLL, % predicted	101.6 ± 20.9	1	107.4 ± 20.2	**< 0.001**

The “current smokers” group consists of the current smokers who were excluded from the lung-healthy group and were filtered for all other exclusion criteria. “Airway obstruction” refers to participants who were excluded from the lung-healthy group based on a Forced Expiratory Volume in 1 s/Forced Vital Capacity ratio below the lower limit of normal, and subsequently filtered for all other exclusion criteria. The percentage of predicted lung and lobar volumes for all groups was calculated using the reference equations (Table 2). Statistical significance was determined through independent two-sided *t*-tests. The adjusted *p*-values were obtained using the Bonferroni method to account for multiple comparisons (24 tests in total). *p*-values larger than 1, which may occur due to the Bonferroni correction, are truncated to 1

*TLV* total lung volume, *LUL* left upper lobe, *LLL* left lower lobe, *RUL* right upper lobe, *RLL* right lower lobe, *RML* right middle lobe. Bold values in Table 3 mean *p* values below 0.05.

## Discussion

In this study, we established reference equations for lung and lobar volumes, based on inspiratory chest CT in a large sample of a general population without lung disease, using linear regression analysis. Age and height were positively associated with lung and lobar volumes, except for the LLL in women. The RLL was consistently the largest lobe, followed by the LUL and LLL, then the RUL and lastly the RML. Upper lobes showed a stronger association with age, indicating they comprise more of the total lung volume as individuals age. Men have higher lung volumes than women at the same height. Overall, age and height explained 7.8–19.9% of the variation in volumes.

Lung volume measurement by CT has the benefit of allowing regional volume assessment, such as lobar volumes, possibly beneficial for treatments like EBV and lung volume reduction. The only other modality capable of achieving this is VP/SPECT, which is much less available [19]. Prior studies, specifically related to lobar volumes, have mostly focused on ventilation or collapsibility of the lobes by comparing scans in in- and expiration [20, 21]. In EBV studies, attention regarding lobar volumes centers on pre- and post-treatment changes [22]. A lower limit for important difference in lobar volume reduction between pre- and post-treatment has been established at 563 mL [23]. This metric does not take into account variations influenced by patient height, age, and sex and may therefore be refined by an approach similar to that employed in the present study. In terms of reference equations for lobar volumes, to our knowledge, only one study previously established such equations at full inspiration based on a cohort of 469 COPDgene participants without COPD (92 never smokers and 377 current or former smokers), incorporating height, sex, and ethnicity [5]. Unlike our study and the GLI reference equations for TLC, they did not observe a significant relationship between age and any of the volumes, possibly attributed to the small cohort size. They did find similar results regarding the relationship to height and a similar intercept [5]. In our study, ethnicity stratification was not performed, given that 97.5% of the participants identified as having Caucasian origins. Similarly to this prior study, we stratified for sex, justified by our finding that women had consistently smaller lobar volumes for the same height compared to men.

The observation that upper lobes had a greater increase in volume with age, as opposed to lower lobes, suggests a potential susceptibility of upper lobes to damage, even among the lung-healthy population. This aligns with research indicating a higher incidence of emphysema and lung cancer in the upper lobes [24, 25]. Therefore, it would be expected that smokers and those with COPD would exhibit relatively larger upper lobes compared to their lower lobes when compared to healthy individuals. In this study, we observed a significant difference between the lung-healthy population and current smokers for every lung or lobar volume, except for the LLL and RML in men and the RLL in men and women. This further supports the idea that the upper lobes may have a greater susceptibility to damage. Another possible explanation, especially in non-smoking individuals, is dysanapsis, which is the mismatch of airway caliber to lung volume [26]. An abnormally high lung volume in combination with a relatively normal caliber airway may result in a measured obstruction, as defined by FEV_1_/FVC.

Regarding CT lung volumes, previous studies primarily compared TLV measurements obtained at inspiration and/or expiration to plethysmography-derived volumes, showing strong correlations [6–14]. Typically, TLC was slightly higher than measured TLV, while RV was lower at expiration [27]. In line with this, a previous study established that there is a substantial discrepancy between the GLI-predicted TLC and TLV, with significantly larger estimated TLC than measured TLV [15]. This difference can only partially be explained by measurement position variations between seated plethysmography and supine CT scans, with a 9.9% smaller volume in the supine position [28].

This study’s strengths lie in its extensive lung-healthy sample of 7306 individuals. Additionally, the current study included a standardized CT scan protocol on third-generation dual-source CT, with very short acquisition time due to high-pitch scanning mode. Previous research has shown consistent CT-based measurements for lobar volumes, with TLV being more reproducible than TLC as measured by body plethysmography [11], which further validates our approach. The reference equations are applicable for assessing lobe-specific hyperinflation in COPD, which is specifically potentially useful in treatments like EBV and lung volume reduction. Furthermore, these equations may be of potential value in assessing other conditions like restrictive pulmonary diseases or surgical planning for lobe removal in lung cancer patients. The application of the derived equations to smokers and subjects with airway obstruction indeed showed higher volumes (especially in the upper lobes). This shows that the equations are capable of pinpointing differences between groups.

### Limitations

The study focused on a regionally specific population from the north of the Netherlands, characterized by above-average height, approximately 5 cm above the WHO growth chart median [29], and predominantly Caucasian ethnicity. Additionally, this cohort consisted of individuals between the ages of 45 and 80. Given that EBV is typically used for severe emphysema patients aged around 50–70, these limitations are acceptable for our purposes. However, it is crucial to recognize that these reference equations may therefore not be universally applicable. It would be important to calibrate these reference equations in other populations.

The explained variance for the lobar and lung volumes was relatively low, ranging from 7.8 to 19.9%. This suggests that other factors not included in the regression analysis play a role in the size of the lobes and lungs. However, these factors are unknown, and no other explanatory factors are used in similar approaches to reference equations, such as the GLI reference equations for TLC, which reported coefficients of variation of over 10% for their models [2]. These unknown factors are likely related to the shape and size of the chest. While sex and height are now used as crude approximations for these factors, they do not fully capture the complexity of individual variations. Genetic factors could also play a role, as well as aspects like fissure integrity.

In Lifelines, plethysmography measurements are not available, which prevents the comparison or correlation of participants’ TLC with their TLV measurements.

## Conclusion

This study establishes reference equations for lobar volumes and total lung volume at inspiratory chest CT by sex, adjusting for age and height in a population of lung-healthy individuals of Northern European descent between the ages of 45 and 80. Lobar volumes were higher in taller individuals and in men and increased with age, especially for upper lobes.

## Supplementary information

Supplementary material

## Abbreviations

COPD: Chronic obstructive pulmonary disease
CT: Computed tomography
EBV: Endobronchial valve
FEV1: Forced expiratory volume in 1 s
FVC: Forced vital capacity
GLI: Global Lung Initiative
LLL: Left lower lobe
LUL: Left upper lobe
RLL: Right lower lobe
RML: Right middle lobe
RSD: Residual standard deviation
RUL: Right upper lobe
TLV: Total lung volume
