# Supplementary material for: Reference formulas for chest CT-derived lobar volumes in the lung-healthy general population
Source: Eur Radiol. 2024 Oct 16;35(5):2912–21. doi: 10.1007/s00330-024-11123-6 (PMC12021944; doi:10.1007/s00330-024-11123-6)
Supplement: Supplementary file 1 — Supplementary material [file 330_2024_11123_MOESM1_ESM.pdf]

Supplement

Imaging signs of respiratory illness on CT:

- Reticulation
- Ground-Glass
- Honey combing
- Micronodules
- Crazy paving
- Bronchiectasis
- Mucous plugging
- Emphysema greater than trace amounts
- Atelectasis
- Consolidation
- Thickened pleura
- Pleural effusion

Smoking-status definitions:

- Never smoking was defined based on self-reported never smoking history and 0 pack years included in the standardized questionnaires.
- Ex smoking was identified as individuals who self-reported quitting smoking without restarting smoking.

- Current smoking was identified as individuals who self-reported smoking and have been smoking within the last month and do not report having quit smoking.

The full smoking related secondary variable definitions can be found at http://wiki-lifelines.web.rug.nl/doku.php?id=smoking_derivatives_v2

Spirometry – lower limit of normal

The lower limit of normal for pulmonary function tests is taken to be equal to the 5th percentile of a healthy non-smoking population. These were calculated automatically per participant based on age/height/weight/sex as per the normalization profiles present in the Welch Allyn SpiroPerfect device and software. The full protocol for spirometry is available for review at http://wiki.lifelines.nl/doku.php?id=pulmonary_function_test
